# Whole blood DNA methylation aging markers predict colorectal cancer survival: a prospective cohort study

**DOI:** 10.1186/s13148-020-00977-4

**Published:** 2020-11-30

**Authors:** Xīn Gào, Yan Zhang, Daniel Boakye, Xiangwei Li, Jenny Chang-Claude, Michael Hoffmeister, Hermann Brenner

**Affiliations:** 1 Division of Clinical Epidemiology and Aging Research, German Cancer Research Center (DKFZ), Im Neuenheimer Feld 581, 69120 Heidelberg, Germany; 2 Division of Cancer Epidemiology, German Cancer Research Center (DKFZ), Im Neuenheimer Feld 581, 69120 Heidelberg, Germany; 3 Medical Faculty of Heidelberg, Heidelberg University, Im Neuenheimer Feld 672, 69120 Heidelberg, Germany; 4 Division of Preventive Oncology, German Cancer Research Center (DKFZ) and National Center for Tumor Diseases (NCT), Im Neuenheimer Feld 460, 69120 Heidelberg, Germany; 5 German Cancer Consortium, German Cancer Research Center (DKFZ), Im Neuenheimer Feld 280, 69120 Heidelberg, Germany

**Keywords:** DNA methylation, Aging, Whole blood, Colorectal cancer, Prognosis, Mortality

## Abstract

**Background:**

Blood DNA methylation-based aging algorithms predict mortality in the general population. We investigated the prognostic value of five established DNA methylation aging algorithms for patients with colorectal cancer (CRC).

**Methods:**

AgeAccelHorvath, AgeAccelHannum, DNAmMRscore, AgeAccelPheno and AgeAccelGrim were constructed using whole blood epi-genomic data from 2206 CRC patients. After a median follow-up of 6.2 years, 1079 deaths were documented, including 596 from CRC. Associations of the aging algorithms with survival outcomes were evaluated using the Cox regression and survival curves. Harrell’s *C*-statistics were computed to investigate predictive performance.

**Results:**

Adjusted hazard ratios (95% confidence intervals) of all-cause mortality for patients in the third compared to the first tertile were 1.66 (1.32, 2.09) for the DNAmMRscore, 1.35 (1.14, 1.59) for AgeAccelPheno and 1.65 (1.37, 2.00) for AgeAccelGrim, even after adjustment for age, sex and stage. AgeAccelHorvath and AgeAccelHannum were not associated with all-cause or CRC-specific mortality. In stage-specific analyses, associations were much stronger for patients with early or intermediate stage cancers (stages I, II and III) than for patients with metastatic (stage IV) cancers. Associations were weaker and less often statistically significant for CRC-specific mortality. Adding DNAmMRscore, AgeAccelPheno or AgeAccelGrim to models including age, sex and tumor stage improved predictive performance moderately.

**Conclusions:**

DNAmMRscore, AgeAccelPheno and AgeAccelGrim could serve as non-invasive CRC prognostic biomarkers independent of other commonly used markers. Further research should aim for tailoring and refining such algorithms for CRC patients and to explore their value for enhanced prediction of treatment success and treatment decisions.

## Introduction

Colorectal cancer (CRC) is one of the leading causes of cancer death, accounting for approximately 9% of the total cancer deaths globally [1]. While declines in CRC mortality rates occurred in Western countries in recent years, CRC mortality rates continue to increase in many middle- and low-income countries [2]. Besides enhanced early detection, enhanced prediction of patients’ prognosis might open new avenues of more effective, personalized treatment strategies to further reduce mortality rates [3]. The tumor-node-metastasis (TNM) staging system is widely utilized to predict CRC prognosis and to guide adjuvant therapy after potential curative surgery. However, the TNM system is not satisfactory in predicting clinical outcomes for patients with intermediate stages [4], and markers that have prognostic value beyond the TNM system are highly desirable.

Research on prognostic markers for CRC patients has largely focused on characteristics of the tumor tissue, whereas less research has been devoted to other indicators of CRC patient prognosis. Recently, a number of studies have disclosed major prognostic value of aging-related changes in methylation of whole blood DNA with respect to mortality in general population cohorts [5–9]. If and to what extent they may also be useful for predicting chances of survival of CRC patients has, to our knowledge, not previously been addressed in large-scale studies. We aimed to evaluate the prognostic value of five recently proposed aging-related algorithms of DNA methylation (DNAm) derived from whole blood DNA with respect to total and CRC-specific mortality in a large cohort of CRC patients from Germany.

## Methods

### Study design and population

Our analysis is based on prospective follow-up of CRC patients recruited in the context of the German DACHS (Darmkrebs: Chancen der Verhütung durch Screening) Study, an ongoing population-based case–control study on CRC. Details of the DACHS study design have been described elsewhere [10–13]. In brief, patients with a first diagnosis of CRC (ICD 10 codes C18-C20) aged at least 30 years (without an upper age limit) are recruited in all of the 22 clinics providing first-line treatment for CRC in the Rhine-Neckar region in Southern Germany. The current analysis includes patients diagnosed in 2003–2010 for whom comprehensive follow-up with respect to survival outcomes was completed and for whom DNA methylation microarray data from blood samples taken at baseline were available.

### Data collection

The patients were recruited by their treating physician during first hospital stay due to CRC and notified to the study center at the German Cancer Research Center after receipt of informed consent. Personal interviews by trained interviewers were scheduled at the earliest possible convenience, either during hospital stay or shortly thereafter at patients’ homes, in which sociodemographic information, medical and lifestyle information was collected using a standardized questionnaire. Comprehensive medical data, including data on patient and tumor characteristics and treatment, were extracted from medical records. Peripheral blood samples were collected after the interview and stored at − 80 °C. The time of blood drawing could be prior (within 2 weeks) to surgery and after surgery including before, during and after adjuvant therapy. Standardized follow-up information on newly diagnosed diseases and recurrences was provided by patients’ physicians 3 and 5 years after diagnosis. Data on vital status, date and cause of death were obtained from local population registers and public health authorities. All patients provided written informed consent. The study was approved by the ethical committees of the Medical Faculty of the University of Heidelberg and the Medical Chambers of Baden-Württemberg and Rhineland-Palatinate.

### DNAm assessment

DNA was extracted from whole blood samples using standard procedures. Whole blood DNA methylation profiles were obtained using the Infinium MethylationEPIC BeadChip Kit that covers over 850,000 CpG sites (Illumina, Inc, San Diego, CA, USA) according to the manufacturer's protocol. We excluded probes with detection *P *value > 0.01 or missing value > 10% from the analysis. Pre-processing and normalization of DNA methylation data were conducted following the pipeline of Lehne et al. [14]. The methylation proportions at each CpG site (beta values) were calculated using normalized intensity values. Leukocyte composition was estimated using Houseman’s algorithms [15].

### DNAm aging algorithms calculation

Table 1 shows basic information on five DNAm aging algorithms, including Horvath’s algorithm [5], Hannum’s algorithm [6], DNAm mortality risk score (DNAmMRscore) [7], DNAmPhenoAge [8] and DNAmGrimAge [9]. Typically, DNAm aging algorithms are constructed by regressing the chronological age or a surrogate measure of biological age on a set of CpG sites from specific tissues using penalized regression analyses, such as LASSO or elastic net regression [16]. Horvath’s algorithm was developed based on 353 CpGs that were related to a transformed version of chronological age [5]. Hannum’s algorithm was built based on 71 age-related CpGs [6]. Unlike Horvath’s algorithm and Hannum’s algorithm, DNAmMRscore, DNAmPhenoAge and DNAmGrimAge were developed by replacing prediction of chronological age with prediction of lifespan and/or surrogate of health span [7–9]. To develop DNAmMRscore, 10 of 58 mortality-related CpG sites were selected by LASSO Cox regression model [7]. DNAmPhenoAge is based on 513 CpGs, which were associated with a phenotypic age, a combination of chronological age and nine biomarkers that reflect the function of liver, kidney, metabolism and immune system [8]. Similarly, AgeAccelGrim was constructed with age, sex as well as 1030 CpGs, which were related to smoking pack-year and seven mortality-related plasma proteins [9].

**Table 1:** Overview of DNA methylation aging algorithms

DNAm aging algorithm	Original study	Tissue	*n*_CpGs_	Surrogate measure of biological age*
AgeAccelHorvath	Horvath et al. [5]	Multiple tissues^#^	353	Calibrated chronological age
AgeAccelHannum	Hannum et al. [6]	Whole blood	71	Chronological age
DNAmMRscore	Zhang et al. [7]	Whole blood	10(8)^§^	All-cause mortality
AgeAccelPheno	Levine et al. [8]	Whole blood	513	9 markers^†^, chronological age
AgeAccelGrim	Lu et al. [9]	Whole blood	1030	7 Plasma proteins^‡^, smoking pack-years

AgeAccel, age acceleration; DNAm, DNA methylation; MRscore, mortality risk score

^*^DNAm aging algorithms are usually constructed by regressing mortality and/or a surrogate measure of biological age on a set of CpG sites

^#^Horvath’s epigenetic clock was originally developed based on CpG sites from DNA of 51 different tissues and cell types. In our study, AgeAccelHorvath was calculated based on CpG sites from DNA of whole blood samples

^§^DNAmMRscore was initially developed based on ten CpG sites, of which two CpG sites are not included in Illumina EPIC microarray data. An adapted formula based on the remaining eight CpG sites has been developed using the data from an external cohort, the German ESTHER cohort

^†^9 markers include albumin, creatinine, serum glucose, C-reactive protein, lymphocyte percent, mean cell volume, red cell distribution width, alkaline phosphatase and white blood cell count

^‡^7 plasma proteins include adrenomedullin, beta-2-microglobulim, cystatin C, growth/differentiation factor 15, leptin (Leptin), plasminogen activator inhibitor-1 and tissue inhibitor metalloproteinases 1

Age acceleration (AgeAccel) is defined as the residual resulting from regressing DNAm algorithms on chronological age [5]. Thus, a positive value of AgeAccel indicates accelerated aging and premature mortality. In this analysis, the age acceleration of Horvath’s algorithm, Hannum’s algorithm, DNAm PhenoAge and DNAm GrimAge were used and denoted by AgeAccelHorvath, AgeAccelHannum, AgeAccelPheno and AgeAccelGrim, respectively. They were computed using an online DNAm aging algorithm calculator (https://dnamage.genetics.ucla.edu) [5]. DNAmMRscore was not transformed to the AgeAccel version since it originally was designed as a predictor of mortality [7]. In addition, two CpGs of the original DNAmMRscore, which had been derived using a 450 K CpG DNA methylation microarray, were not included in the EPIC microarray data. We thus developed an equation (as follows) of constructing DNAmMRscore based on eight CpGs by regressing the original DNAmMRscore of ten CpGs on the remaining eight CpGs in the 450 K microarray data of the German ESTHER Study [17], which had been used to develop the DNAmMRscore [7].

1 $$\begin{aligned} {\text{DNAmMRscore}}&= - 0.36909 - 1.09957 \times cg01612140\\ & \quad - 1.65446 \times cg05575921 + 3.12883 \times cg08362785\\ & \quad - 0.22268 \times cg10321156 - 0.30369 \times cg14975410 \\ & \quad - 0.31940 \times cg19572487 - 3.39726 \times cg24704287\\ & \quad - 1.93238 \times cg25983901 \\ \end{aligned}$$

### Statistical methods

The correlations among AgeAccelHorvath, AgeAccelHannum, DNAmMRscore, AgeAccelPheno and AgeAccelGrim were assessed with Pearson correlation coefficients and scatter plots. The distribution of the DNAm aging algorithms was described by median and interquartile range (IQR) and compared across categorical baseline characteristics of the study population by Kruskal–Wallis test.

Cox proportional hazards regression accounting for delayed entry was used to assess the associations of DNAm aging algorithms [per standard deviation (SD) increase and classified in tertiles] with all-cause mortality (or overall survival) and CRC-specific mortality (or CRC-specific survival). In addition, competing risk was considered in the analysis for CRC-specific mortality. The Schoenfeld Residuals method was applied to test if the algorithms violate the assumption of Cox regression. A “clinical model” was performed as the main model adjusting for the factors that are easily obtained in clinical settings, including chronological age, sex, stage, measurement batch and leukocyte composition (Houseman’s algorithms). Furthermore, stage-specific HRs and survival curves with adjustment for age, sex, batch and leukocyte composition were used to assess whether the association between DNAm markers and CRC prognosis differs depending on tumor stages. Tests for interaction were carried out by setting variable cross-product terms of DNAm aging algorithm with stage in the model. The difference between survival curves was evaluated using the G-rho family of tests.

Sensitivity analyses were performed to investigate the association between DNAm aging algorithms and CRC prognosis with a more comprehensive adjustment for the variables that are shown in Table 2, including age, sex, batch, leukocyte composition, tumor stage, body mass index (BMI, kg/m^2^), smoking status (never, former and current smokers), alcohol consumption (gram of ethanol per day), tumor subsite and Charlson comorbidity index (CCI) score that was calculated from comorbidities at the time of CRC diagnosis [18]. Additionally, to exclude the influence of chemotherapy and/or radiotherapy on DNAm markers, we assessed the association between the DNAm and CRC prognosis among patients who had not received any chemotherapy or radiotherapy during the follow-up.

**Table 2:** Clinical characteristics at baseline in the DACHS study

Baseline Characteristics	Values
Sex, *n* (%)	
Women	910 (41.2)
Men	1296 (58.8)
Age at diagnosis, *n* (%)	
33 ≤–< 55 years	238 (10.8)
55 ≤–< 65 years	479 (21.7)
65 ≤–< 75 years	776 (35.2)
75 ≤–≤ 96 years	713 (32.3)
Tumor stage, *n* (%)*	
I	400 (18.2)
II	760 (34.6)
III	726 (33.1)
IV	309 (14.1)
Leukocyte composition, Median (IQR)^#^	
CD4 + T cells	0.12 (0.07, 0.17)
CD8 + T cells	0.03 (0.004, 0.06)
NK cells	0.07 (0.04, 0.11)
B cells	0.04 (0.03, 0.06)
Monocytes	0.08 (0.06, 0.10)
Granulocytes	0.65 (0.56, 0.74)
Charlson comorbidity index, *n* (%)	
0 (no comorbidity)	1282 (58.1)
1 (mild comorbidity)	479 (21.7)
2 (moderate comorbidity)	264 (12.0)
3+ (severe comorbidity)	182 (8.2)
Tumor sub-site, *n* (%)*	
Proximal colon^†^	796 (36.1)
Distal colon^‡^	738 (33.5)
Rectum	670 (30.4)
BMI at diagnosis, *n* (%)*	–
< 25 kg/m^2^	834 (38.0)
25 ≤–< 30 kg/m^2^	932 (42.4)
≥ 30 kg/m^2^	430 (19.6)
Alcohol consumption, *n* (%)*	–
Abstainer	380 (17.3)
Female: < 20 g/day; Male: < 40 g/day	1559 (70.9)
Female: ≥ 20 g/day; Male: ≥ 40 g/day	260 (11.8)
Smoking status, *n* (%)*	
Never	907 (41.1)
Former	948 (43.0)
Current	350 (15.9)

BMI, body mass index; char, characteristics; IQR, interquartile range

^*^Numbers do not add up to 2206 because of missing data:11 missing values for tumor stage, 10 missing values for BMI, 7 missing values for alcohol consumption and 2 missing values for smoking status. Complete case analysis was applied when adjusting for these variables

^#^Leukocyte composition was estimated by Houseman’s method

^†^The proximal colon includes the cecum, the ascending colon and the transverse colon

^‡^The distal colon includes the descending colon (the left side of the colon) and the sigmoid colon

Predictive accuracy and discriminating ability of DNAm aging algorithms were evaluated using Harrell's concordance statistics (*C*-statistics) and were compared with age, sex and stage. A *C*-statistic value of 0.5 suggests no discrimination, and 1.0 indicates perfect discrimination.

Hazard ratios and Harrell’s *C*-statistics were derived using the PROC PHREG in SAS version 9.4 (SAS Institute, Cary, NC). Correlation matrix and adjusted survival curves were produced using the R 3.6.0 with the packages *corrplot* and *survminer*, respectively [19]. Statistical significance was defined by *P* < 0.05 in two-sided testing.

## Results

### Clinical characteristics of study population

We included 2206 eligible patients diagnosed with CRC, of whom 18.1%, 34.4%, 32.9% and 14.0% were diagnosed in stage I, II, III and IV, respectively. Over a median of 6.2 years (IQR 3.7–10.1) of follow-up, a total of 1079 deaths occurred, including 596 deaths due to CRC.

Table 2 describes baseline characteristics of the study population, which included more men (58.8%) than women and had a median age of 69 years. Most patients were diagnosed in either stage II (34.6%) or stage III (33.1%), and more than 40% had relevant comorbidity (CCI > 0). The distribution of AgeAccelHorvath, AgeAccelHannum, DNAmMRscore, AgeAccelPheno and AgeAccelGrim according to categorical baseline characteristics is presented in Additional file 1: Table S1. The levels of all DNAm aging algorithms were higher in females, smokers, those consuming more alcohol and patients with higher CCI and advanced stage CRC. Additional file 1: Fig. S1 presents correlations of AgeAccelHannum, DNAmMRscore, AgeAccelPheno and AgeAccelGrim with leukocyte composition. Levels of DNAm aging algorithms did not vary by the year of blood sampling (Additional file 1: Fig. S2).

### Correlation among DNAm aging algorithms

All DNAm aging algorithms were statistically significantly correlated with each other, as shown in Additional file 1: Fig. S3. DNAmMRscore showed a moderate positive correlation with AgeAccelHannum (*ρ* = 0.46), AgeAccelPheno (*ρ* = 0.46) and AgeAccelGrim (*ρ* = 0.63), but a weaker correlation with AgeAccelHorvath (*ρ* = 0.14).

### Association of DNAm aging algorithms with CRC prognosis

**Fig. 1:**
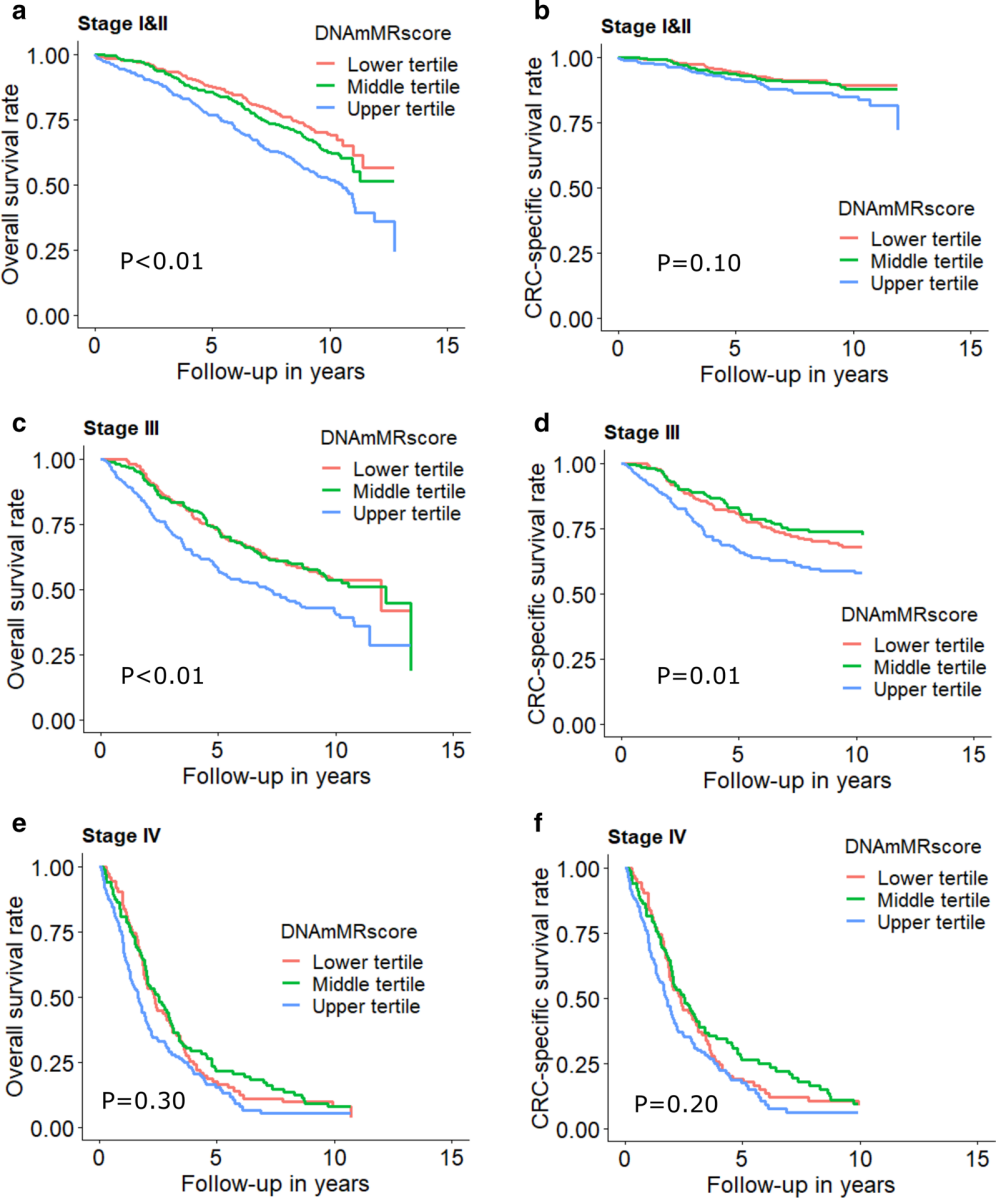
Stage-specific survival curves for overall and cancer-specific survival of CRC patients by tertiles of DNAmMRscore. **a** Overall and **b** CRC-specific survival curve among stage I and II patients; **c** overall and **d** CRC-specific survival curve among stage III; **e** overall and **f** CRC-specific survival curve among stage IV. Stage-specific survival curves were adjusted for age, sex, batch and leukocyte composition (Houseman’s algorithm)

**Fig. 2:**
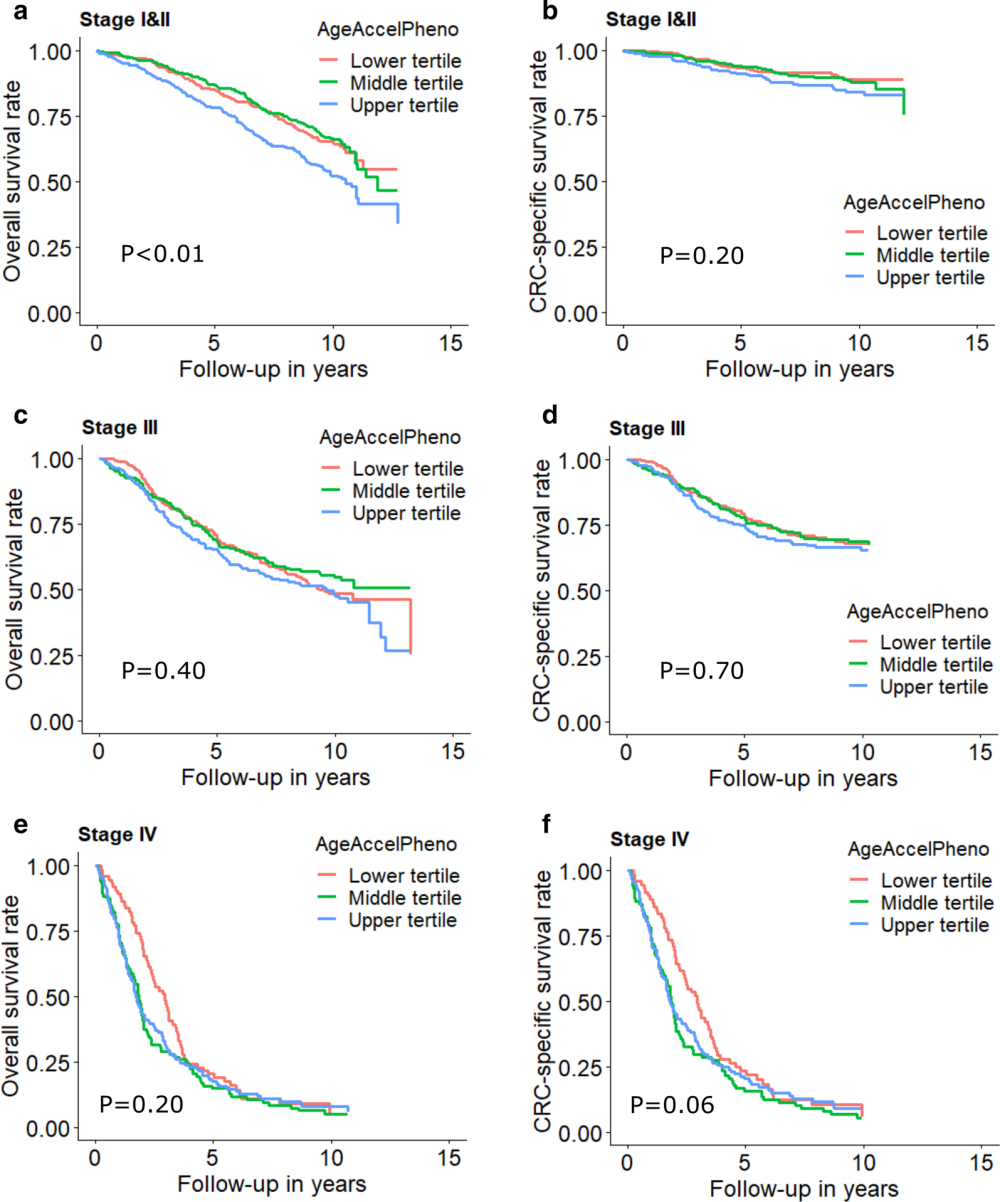
Stage-specific survival curves for overall and cancer-specific survival of CRC patients by tertiles of AgeAccelPheno. **a** Overall and **b** CRC-specific survival curve among stage I and II patients; **c** overall and **d** CRC-specific survival curve among stage III; **e** overall and **f** CRC-specific survival curve among stage IV. Stage-specific survival curves were adjusted for age, sex, batch and leukocyte composition (Houseman’s algorithm)

**Fig. 3:**
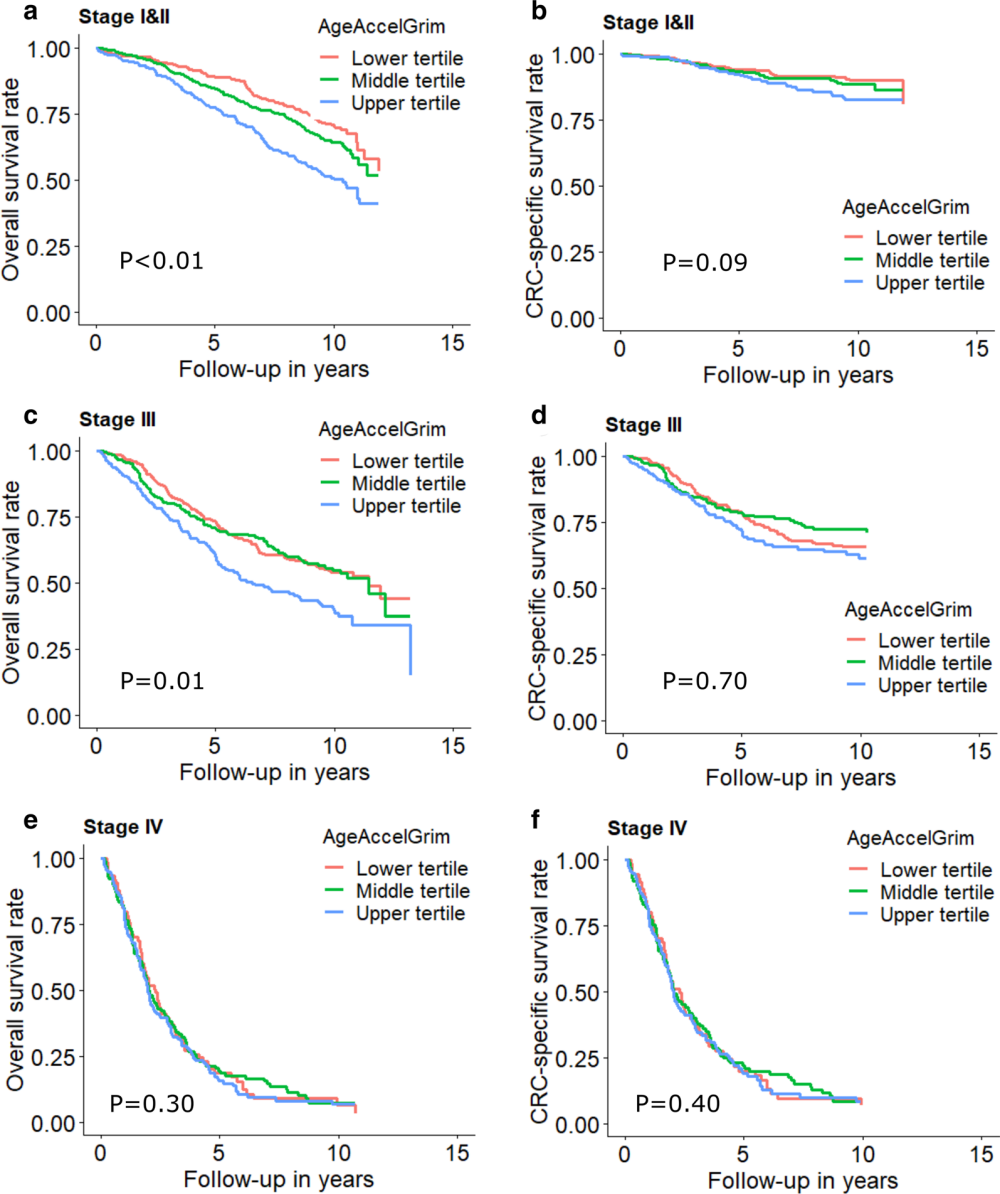
Stage-specific survival curves for overall and cancer-specific survival of CRC patients by tertiles of AgeAccelGrim. **a** Overall and **b** CRC-specific survival curve among stage I and II patients; **c** overall and **d** CRC-specific survival curve among stage III; **e** overall and **f** CRC-specific survival curve among stage IV. Stage-specific survival curves were adjusted for age, sex, batch and leukocyte composition (Houseman’s algorithm)

Table 3 shows the association between DNAm aging algorithms and all-cause mortality of CRC patients. In the analyses including patients with any stage, we observed marginal non-significant associations for AgeAccelHorvath and AgeAccelHannum and statistically significant associations for DNAmMRscore, AgeAccelPheno and AgeAccelGrim. HRs (95%CIs) were 1.17 (0.99, 1.38), 1.12 (0.94, 1.33), 1.66 (1.32, 2.09), 1.35 (1.14, 1.59) and 1.65 (1.37, 2.00) for the association of all-cause mortality with upper (vs. lower) tertiles of AgeAccelHorvath, AgeAccelHannum, DNAmMRscore, AgeAccelPheno and AgeAccelGrim, respectively. In stage-specific analysis, as shown in Table 3 and Figs. 1, 2 and 3, associations of DNAm aging algorithms and overall survival attenuated with increased severity of CRC. Survival was worst among the patients with highest levels of DNAm aging algorithms for early and intermediate stage. Among stage IV patients, medium levels of AgeAccelPheno were associated with highest risk of mortality (Fig. 2). However, the interaction between the algorithms and stages was not statistically significant.

**Table 3:** Associations of DNA methylation aging markers with all-cause mortality

Markers	Categories	All stages		Stage I and II		Stage III		Stage IV		*P* for interaction
		*n*_death_/*n*_cases_	HR (95% CI)*	*n*_death_/*n*_cases_	HR (95% CI)^#^	*n*_death_/*n*_cases_	HR (95% CI)^#^	*n*_death_/*n*_cases_	HR (95% CI)^#^	
AgeAccelHorvath	Tertile 1	361/736	1.00 (Ref.)	135/364	1.00 (Ref.)	124/260	1.00 (Ref.)	100/107	1.00 (Ref.)	–
	Tertile 2	339/736	1.01 (0.86, 1.18)	138/402	0.97 (0.76, 1.24)	109/236	0.95 (0.73, 1.25)	87/92	1.14 (0.83, 1.55)	–
	Tertile 3	379/734	1.17 (0.99, 1.38)	163/393	**1.32 (1.04, 1.38)**	117/230	1.18 (0.90, 1.55)	98/110	0.98 (0.71, 1.34)	–
	Per SD increase	1079/2206	1.06 (0.99, 1.13)	436/1159	1.10 (0.99, 1.21)	350/726	1.10 (0.99, 1.23)	285/309	0.96 (0.84, 1.10)	0.139
AgeAccelHannum	Tertile 1	327/736	1.00 (Ref.)	128/386	1.00 (Ref.)	124/268	1.00 (Ref.)	74/80	1.00 (Ref.)	–
	Tertile 2	340/736	0.93 (0.78, 1.09)	126/387	0.89 (0.69, 1.15)	109/235	0.93 (0.70, 1.24)	103/111	0.86 (0.60, 1.24)	–
	Tertile 3	412/734	1.12 (0.94, 1.33)	182/386	1.18 (0.91, 1.53)	117/223	1.14 (0.84, 1.55)	108/118	0.91 (0.62, 1.34)	–
	Per SD increase	1079/2206	1.06 (0.98, 1.14)	436/1159	1.05 (0.93, 1.17)	350/726	1.11 (0.98, 1.26)	285/309	1.00 (0.86, 1.17)	0.475
DNAmMRscore	Tertile 1	275/736	1.00 (Ref.)	102/410	1.00 (Ref.)	97/242	1.00 (Ref.)	76/84	1.00 (Ref.)	
	Tertile 2	346/736	1.07 (0.89, 1.30)	147/399	1.27 (0.94, 1.70)	104/231	0.97 (0.71, 1.34)	92/102	0.89 (0.61, 1.29)	
	Tertile 3	458/734	**1.66 (1.32, 2.09)**	187/350	**2.01 (1.42, 2.85)**	149/253	**1.64 (1.13, 2.38)**	117/123	1.33 (0.87, 2.04)	–
	Per SD increase	1079/2206	**1.34 (1.20, 1.49)**	436/1159	**1.50 (1.28, 1.76)**	350/726	**1.39 (1.18, 1.64)**	285/309	1.09 (0.90, 1.33)	0.616
AgeAccelPheno	Tertile 1	318/736	1.00 (Ref.)	122/390	1.00 (Ref.)	123/263	1.00 (Ref.)	71/80	1.00 (Ref.)	–
	Tertile 2	323/736	1.02 (0.86, 1.20)	126/395	0.89 (0.68, 1.15)	99/234	0.92 (0.69, 1.22)	96/102	**1.43 (1.02, 2.01)**	–
	Tertile 3	438/734	**1.35 (1.14, 1.59)**	188/374	**1.50 (1.17, 1.92)**	128/229	1.28 (0.98, 1.69)	118/127	1.31 (0.93, 1.86)	–
	Per SD increase	1079/2206	**1.18 (1.10, 1.27)**	436/1159	**1.22 (1.11, 1.35)**	350/726	**1.20 (1.07, 1.35)**	285/309	1.12 (0.97, 1.30)	0.459
AgeAccelGrim	Tertile 1	286/736	1.00 (Ref.)	98/405	1.00 (Ref.)	119/258	1.00 (Ref.)	68/72	1.00 (Ref.)	–
	Tertile 2	347/736	1.13 (0.94, 1.34)	144/381	**1.34 (1.00, 1.78)**	106/246	0.98 (0.73, 1.32)	95/104	0.95 (0.66, 1.37)	–
	Tertile 3	446/734	**1.65 (1.37, 2.00)**	194/373	**2.30 (1.69, 3.13)**	125/222	**1.63 (1.19, 2.22)**	122/133	1.01 (0.71, 1.45)	–
	Per SD increase	1079/2206	**1.35 (1.24, 1.47)**	436/1159	**1.48 (1.31, 1.66)**	350/726	**1.24 (1.08, 1.43)**	285/309	**1.23 (1.05, 1.44)**	0.082

AgeAccel, age acceleration; CI, confidence interval; DNAm, DNA methylation; HR, hazard ratio; MRscore, mortality risk score

Numbers printed in bold: statistically significantly different from 1 (*P* < 0.05)

^*^Overall HR was adjusted for age, sex, batch effects, tumor stage and leukocyte composition (Houseman’s algorithm)

^#^Stage-specific HR was adjusted for age, sex, batch effects and leukocyte composition (Houseman’s algorithm)

As shown in Table 4, associations of higher DNAm aging algorithms with poorer survival were weaker for CRC-specific survival than for overall survival. HRs (95% CIs) for the comparison of the upper tertile with the lower tertile of AgeAccelHorvath, AgeAccelHannum, DNAmMRscore, AgeAccelPheno and AgeAccelGrim were 1.17 (0.94, 1.46), 1.06 (0.83, 1.36), 1.54 (1.11, 2.15), 1.25 (0.98, 1.59) and 1.28 (0.84, 1.93), respectively. Table 4 and Figs. 1, 2 and 3 show that only AgeAccelHorvath was statistically significantly associated with CRC-specific mortality among stage I and II patients. Among stage III patients, the associations were statistically significant for DNAmMRscore and AgeAccelPheno. Among stage IV patients, AgeAccelGrim showed a marginally significant association with CRC-specific mortality.

**Table 4:** Associations of DNA methylation aging markers with CRC-specific mortality

Markers	Categories	All stages		Stage I and II		Stage III		Stage IV		*P* for interaction
		*n*_death_/*n*_cases_	HR (95% CI)*	*n*_death_/*n*_cases_	HR (95% CI)^#^	*n*_death_/*n*_cases_	HR (95% CI)^#^	*n*_death_/*n*_cases_	HR (95% CI)^#^	
AgeAccelHorvath	Tertile 1	203/732	1.00 (Ref.)	34/362	1.00 (Ref.)	75/257	1.00 (Ref.)	93/105	1.00 (ref.)	–
	Tertile 2	184/731	1.03 (0.83, 1.28)	32/401	0.88 (0.54, 1.45)	69/234	1.02 (0.73, 1.42)	82/91	1.11 (0.80, 1.52)	–
	Tertile 3	209/728	1.17 (0.94, 1.46)	49/390	**1.67 (1.06, 2.65)**	67/230	1.17 (0.83, 1.66)	92/109	0.95 (0.68, 1.31)	–
	Per SD increase	596/2191	1.04 (0.95, 1.13)	115/1153	1.17 (0.97, 1.41)	211/721	1.07 (0.93, 1.24)	267/305	0.94 (0.81, 1.08)	0.256
AgeAccelHannum	Tertile 1	173/732	1.00 (Ref.)	28/384	1.00 (Ref.)	77/266	1.00 (Ref.)	68/78	1.00 (ref.)	–
	Tertile 2	204/731	0.93 (0.73, 1.17)	45/386	1.46 (0.89, 2.40)	61/234	0.82 (0.57, 1.18)	98/110	0.88 (0.60, 1.29)	–
	Tertile 3	219/728	1.06 (0.83, 1.36)	42/383	1.24 (0.72, 2.12)	73/221	1.13 (0.78, 1.65)	101/117	0.90 (0.60, 1.35)	–
	Per SD increase	596/2191	1.07 (0.97, 1.19)	115/1153	1.05 (0.84, 1.31)	211/721	1.10 (0.94, 1.29)	267/305	1.02 (0.87, 1.20)	0.258
DNAmMRscore	Tertile 1	166/733	1.00 (Ref.)	31/409	1.00 (Ref.)	63/241	1.00 (Ref.)	72/83	1.00 (ref.)	
	Tertile 2	176/729	0.90 (0.68, 1.17)	38/395	1.07 (0.61, 1.86)	53/229	0.81 (0.53, 1.25)	84/101	0.82 (0.56, 1.21)	
	Tertile 3	254/729	**1.54 (1.11, 2.15)**	46/349	1.38 (0.71, 2.66)	95/251	1.61 (1.00, 2.57)	111/121	1.21 (0.77, 1.88)	–
	Per SD increase	596/2191	**1.30 (1.11, 1.53)**	115/1153	1.34 (0.99, 1.81)	211/721	**1.36 (1.10, 1.67)**	267/305	1.06 (0.86, 1.29)	0.041
AgeAccelPheno	Tertile 1	168/732	1.00 (Ref.)	30/389	1.00 (Ref.)	72/262	1.00 (Ref.)	65/78	1.00 (ref.)	–
	Tertile 2	196/731	1.20 (0.96, 1.51)	40/394	1.08 (0.66, 1.78)	63/231	1.01 (0.70, 1.45)	93/101	1.51 (1.06, 2.14)	**–**
	Tertile 3	232/728	1.25 (0.98, 1.59)	45/370	1.38 (0.84, 2.29)	76/228	1.26 (0.89, 1.79)	109/126	1.26 (0.88, 1.81)	–
	Per SD increase	596/2191	**1.15 (1.04, 1.27)**	115/1153	1.18 (0.97, 1.42)	211/721	**1.22 (1.06, 1.42)**	267/305	1.08 (0.92, 1.25)	0.281
AgeAccelGrim	Tertile 1	175/732	1.00 (Ref.)	32/404	1.00 (Ref.)	80/257	1.00 (Ref.)	63/70	1.00 (ref.)	–
	Tertile 2	188/731	0.92 (0.73, 1.17)	38/379	1.14 (0.68, 1.93)	60/245	0.79 (0.54, 1.15)	90/103	0.92 (0.64, 1.34)	–
	Tertile 3	233/728	1.28 (0.84, 1.93)	45/370	1.53 (0.85, 2.73)	71/219	1.19 (0.80, 1.77)	114/132	0.94 (0.65, 1.37)	–
	Per SD increase	596/2191	**1.26 (1.09, 1.45)**	115/1153	1.24 (0.99, 1.56)	211/721	1.10 (0.92, 1.31)	267/305	**1.18 (1.00, 1.40)**	0.036

AgeAccel, age acceleration; CI, confidence interval; DNAm, DNA methylation; HR, hazard ratio; MRscore, mortality risk score

Numbers printed in bold: statistically significantly different from 1 (*P* < 0.05)

^*^Overall HR was adjusted for age, sex, batch effects, tumor stage and leukocyte composition (Houseman’s algorithm)

^#^Stage-specific HR was adjusted for age, sex, batch effects and leukocyte composition (Houseman’s algorithm)

God Additional file 1: Tables S2 and S3 show that additional adjustments for BMI, smoking status, alcohol consumption, tumor subsite and CCI changed the association of DNAm aging algorithms with all-cause mortality and CRC-specific mortality only slightly. Additional file 1: Table S4 shows that the associations of DNAmMRscore, AgeAccelPheno and AgeAccelGrim with both outcomes were stronger among patients who received surgery only. AgeAccelHorvath was statistically significantly associated with all-cause mortality, but not with CRC-specific mortality.

### Predictive utility of DNAmMRscore, AgeAccelPheno and AgeAccelGrim

Table 5 presents the discrimination ability of various combinations of CRC prognostic factors, including age, sex, stage, DNAmMRscore, AgeAccelPheno and AgeAccelGrim. The performance of prediction was moderately improved after adding DNAm aging algorithms in models including age, sex and stage. For all-cause mortality, models including AgeAccelGrim showed tentatively stronger predictive ability than the others among patients of all stages and in patients with stages I and II or III. For CRC-specific mortality, similar improvements in predictive ability were achieved by adding either one of the three algorithms to the models. Moreover, a model of combining DNAmMRscore, AgeAccelPheno and AgeAccelGrim did not significantly improve the predictive performance compared with the single algorithm model (data not shown).

**Table 5:** Harrell’s *C*-statistics (95% CI) for all-cause mortality and CRC-specific mortality prediction

Outcomes	Models	All stages	Stage I and II	Stage III	Stage IV
All-cause mortality	Common predictors*	0.739 (0.723, 0.754)	0.693 (0.667, 0.719)	0.653 (0.622, 0.683)	0.557 (0.520, 0.594)
	+DNAmMRscore^#^	0.747 (0.732, 0.762)	0.709 (0.684, 0.735)	0.668 (0.638, 0.698)	0.592 (0.554, 0.629)
	+AgeAccelPheno^#^	0.746 (0.731, 0.761)	0.708 (0.683, 0.734)	0.662 (0.632, 0.692)	0.587 (0.552, 0.623)
	+AgeAccelGrim^#^	0.754 (0.740, 0.769)	0.725 (0.700, 0.749)	0.671 (0.641, 0.700)	0.589 (0.551, 0.627)
CRC-specific mortality	Common predictors*	0.809 (0.792, 0.825)	0.612 (0.559, 0.666)	0.608 (0.568, 0.648)	0.557 (0.519, 0.595)
	+DNAmMRscore^#^	0.815 (0.798, 0.831)	0.633 (0.582, 0.685)	0.635 (0.595, 0.675)	0.589 (0.551, 0.627)
	+AgeAccelPheno^#^	0.813 (0.797, 0.829)	0.636 (0.584, 0.688)	0.621 (0.582, 0.660)	0.586 (0.549, 0.623)
	+AgeAccelGrim^#^	0.814 (0.798, 0.830)	0.646 (0.596, 0.697)	0.626 (0.586, 0.665)	0.584 (0.545, 0.623)

AgeAccel, age acceleration; CI, confidence interval; MRscore, DNA methylation mortality risk score

^*^Common predictors include age, sex, tumor stage for overall *C*-statistics, and age and sex for stage-specific *C*-statistics

^#^Models include common predictors and the corresponding DNA methylation aging algorithm

## Discussion

To our knowledge, this study is the first to investigate longitudinal association of five frequently used DNAm aging algorithms with CRC prognosis. Of the five algorithms, DNAmMRscore, AgeAccelPheno and AgeAccelGrim were positively associated with all-cause and CRC-specific mortality. Associations were strongest for DNAmMRscore and were generally stronger for all-cause mortality than for CRC-specific mortality. Adding either of DNAmMRscore, AgeAccelPheno or AgeAccelGrim to models including age, sex and stage moderately increased prognostic performance with respect to either all-cause mortality or CRC-specific mortality within all stages, including stage IV.

Previous studies have shown that Horvath’s and Hannum’s algorithms are statistically significantly associated with all-cause mortality in older general populations [20–23]. Consistent with our findings, DNAmMRsocre, PhenoAge and GrimAge outperformed the first generation of DNAm aging algorithms regarding mortality prediction [8, 9, 24–26]. Few studies have focused on the prognostic values of DNAm aging algorithms among cancer patients. Dugué and colleagues compared different variations of Horvath’s and Hannum’s algorithms and concluded that the increased age acceleration was associated with higher cancer mortality [27]. Moreover, Zheng et al. observed a significantly positive association of Horvath’s algorithm with overall survival of CRC for the comparison of age acceleration group and age deceleration group, which is not supported by our study [28]. In Zheng’s analysis, the Cox model was adjusted for only tumor stage and molecular subtype, which may not be sufficient to exclude confounding due to age, sex and leukocyte composition alteration.

DNAmMRscore, AgeAccelPheno and AgeAccelGrim were modestly correlated with each other in our study. Unlike other algorithms, DNAmMRscore is explicitly trained to predict Mortality. It is developed based on much fewer CpG sites that were related to all-mortality, severe conditions and smoking [7]. More clinical and/or lifestyle characteristics were considered in the development of AgeAccelPheno and AgeAccelGrim. As for AgeAccelPheno, chronological age and nine mortality-related clinical markers such as C-reactive protein were integrated and were regressed on DNAm data [8]. Finally, AgeAccelGrim was computed using the methylation pattern of CpG sites, which were associated with seven plasma proteins, smoking pack-year and all-cause mortality [9]. The significant correlation of DNAmMRscore, AgeAccelPheno and AgeAccelGrim with comorbidity suggests that the predictive power for CRC prognosis can be improved by regressing clinical outcomes and biomarkers on DNAm data in the process of CpG sites selection. In other words, DNAmMRscore, AgeAccelPheno and AgeAccelGrim were likely to capture pathophysiological information in the prediction of mortality risk among CRC patients. Although DNAmMRscore, AgeAccelPheno and AgeAccelGrim showed similar predictive performance regarding CRC prognosis, DNAmMRscore achieved such prognostic performance with much fewer CpG sites.

Even though there has been substantial improvement in the prognosis of patients with CRC over the last decades, it remains challenging to stratify patients with specific CRC stages and to make decisions on treatments from which they can benefit most [29]. Although there has been intensive search for blood-based biomarkers with prognostic or predictive ability, few of them retained their prognostic relevance after adjustment for or stratification by CRC stage. Our study showed that DNAm aging algorithms, especially DNAmMRscore, AgeAccelPheno and AgeAccelGrim, were associated with overall survival and disease-specific survival among patients with CRC, independent of age, sex and stage. Therefore, a combination of those DNAm aging algorithms with other clinical factors, such as age, sex and stage, may have the potential to enhance judgment of patients’ prognosis and to improve patient management in clinical practice. However, the associations between DNAm markers and CRC prognosis were weak and mostly statistically non-significant among patients with advanced (stage IV) CRC, among whom prognosis is generally extremely poor. Also, case numbers were smallest in this group which limited statistical power to detect possible associations. Sample size limitations also prohibited in-depth analyses on potential use of the DNAm aging algorithms for predicting success of specific therapies within CRC stages which should be addressed in further, even much larger studies.

Besides potential use for prognostic classification or prediction of treatment success, DNAm aging algorithms can be utilized to explore potential mechanisms and/or synergies underlying the relationship between aging and tumor progression in CRC patients. While our study was the first to demonstrate associations of composite DNAm aging algorithms with CRC prognosis further work should address in more detail which components of the algorithms or which other DNAm markers might be most predictive for CRC prognosis and treatment success, and elucidate in more detail the underlying biological mechanisms. Further studies are also needed to develop novel prognostic DNAm markers and algorithms that are more specific to CRC.

The strengths of this study include the prospective design, large case numbers, long-term follow-up, the well-recorded causes of death, detailed information on pathological data and treatment data. The large sample size allowed detecting moderate size associations which might not be observed in smaller studies. There are also potential limitations that are worth noting. First, surgery, chemo- and radiotherapy administration could affect leukocyte distribution and subsequently have an impact on DNAm levels. Therefore, the leukocyte composition was adjusted for in all Cox regression models to minimize the bias. Sensitivity analyses were performed to investigate the potential bias caused by the timing of blood sampling relative to treatment. Similar results were observed among the patients who received surgery only (Additional file 1: Table S4). Moreover, results barely changed after additionally adjusting the Cox regression model for the timing of blood sampling relative to treatment (data not shown). Second, even though we thoroughly adjusted for potential confounders, residual confounding cannot be completely excluded because of the observational nature of our study. Third, the relatively smaller number of CRC-specific deaths limited the statistical power; therefore, further studies with larger sample size are needed. Last, we investigated a Caucasian population. Caution is therefore required when generalizing the findings to non-Caucasian populations.

In conclusion, DNAmMRscore, AgeAccelPheno and AgeAccelGrim, which incorporate clinical biomarkers and/or features, showed a strong positive association with all-cause mortality among patients with CRC, even within specific CRC stages. They have slight prognostic value beyond age, sex and stage. Further research should address the potential of refinement of DNAm algorithms for predicting prognosis and explore the value of such refined algorithms for predicting success of specific treatments, which may contribute to paving the way for guiding therapeutic decision as well as drug development.

## Supplementary information

**Additional file 1:** Supplementary tables and figures.

## Data Availability

The datasets generated and/or analyzed during the current study are not made publicly available due to ethical and data security requirements but can be made available for researchers on the basis of a research proposal (to be submitted to the corresponding author).

## References

[1] Bray F, Ferlay J, Soerjomataram I, Siegel RL, Torre LA, Jemal A (2018). Global cancer statistics 2018: GLOBOCAN estimates of incidence and mortality worldwide for 36 cancers in 185 countries. CA Cancer J Clin.

[2] Arnold M, Sierra MS, Laversanne M, Soerjomataram I, Jemal A, Bray F (2017). Global patterns and trends in colorectal cancer incidence and mortality. Gut.

[3] Brenner H, Chen C (2018). The colorectal cancer epidemic: challenges and opportunities for primary, secondary and tertiary prevention. Br J Cancer.

[4] Schneider NI, Langner C (2014). Prognostic stratification of colorectal cancer patients: current perspectives. Cancer Manag Res.

[5] Horvath S (2013). DNA methylation age of human tissues and cell types. Genome Biol.

[6] Hannum G, Guinney J, Zhao L, Zhang L, Hughes G, Sadda S (2013). Genome-wide methylation profiles reveal quantitative views of human aging rates. Mol Cell.

[7] Zhang Y, Wilson R, Heiss J, Breitling LP, Saum KU, Schottker B (2017). DNA methylation signatures in peripheral blood strongly predict all-cause mortality. Nat Commun.

[8] Levine ME, Lu AT, Quach A, Chen BH, Assimes TL, Bandinelli S (2018). An epigenetic biomarker of aging for lifespan and healthspan. Aging-US.

[9] Lu AT, Quach A, Wilson JG, Reiner AP, Aviv A, Raj K (2019). DNA methylation GrimAge strongly predicts lifespan and healthspan. Aging-US.

[10] Brenner H, Chang-Claude J, Seiler CM, Rickert A, Hoffmeister M (2011). Protection from colorectal cancer after colonoscopy: a population-based, case-control study. Ann Intern Med.

[11] Brenner H, Chang-Claude J, Jansen L, Knebel P, Stock C, Hoffmeister M (2014). Reduced risk of colorectal cancer up to 10 years after screening, surveillance, or diagnostic colonoscopy. Gastroenterology.

[12] Hoffmeister M, Jansen L, Rudolph A, Toth C, Kloor M, Roth W (2015). Statin use and survival after colorectal cancer: the importance of comprehensive confounder adjustment. J Natl Cancer Inst.

[13] Carr PR, Weigl K, Edelmann D, Jansen L, Chang-Claude J, Brenner H, Hoffmeister M (2020). Estimation of absolute risk of colorectal cancer based on healthy lifestyle, genetic risk, and colonoscopy status in a population-based study. Gastroenterology.

[14] Lehne B, Drong AW, Loh M, Zhang W, Scott WR, Tan ST (2015). A coherent approach for analysis of the Illumina HumanMethylation450 BeadChip improves data quality and performance in epigenome-wide association studies. Genome Biol.

[15] Houseman EA, Accomando WP, Koestler DC, Christensen BC, Marsit CJ, Nelson HH (2012). DNA methylation arrays as surrogate measures of cell mixture distribution. BMC Bioinform.

[16] Horvath S, Raj K (2018). DNA methylation-based biomarkers and the epigenetic clock theory of ageing. Nat Rev Genet.

[17] Gào X, Wilsgaard T, Jansen EH, Holleczek B, Zhang Y, Xuan Y (2019). Pre-diagnostic derivatives of reactive oxygen metabolites and the occurrence of lung, colorectal, breast and prostate cancer: An individual participant data meta-analysis of two large population-based studies. Int J Cancer.

[18] Boakye D, Jansen L, Schneider M, Chang-Claude J, Hoffmeister M, Brenner H (2019). Personalizing the prediction of colorectal cancer prognosis by incorporating comorbidities and functional status into prognostic nomograms. Cancers.

[19] R Core Team. R: a language and environment for statistical computing. R Foundation for Statistical Computing, Vienna; 2019. http://www.r-project.org/index.html.

[20] Marioni RE, Shah S, McRae AF, Chen BH, Colicino E, Harris SE (2015). DNA methylation age of blood predicts all-cause mortality in later life. Genome Biol.

[21] Chen BH, Marioni RE, Colicino E, Peters MJ, Ward-Caviness CK, Tsai PC (2016). DNA methylation-based measures of biological age: meta-analysis predicting time to death. Aging-US.

[22] Christiansen L, Lenart A, Tan Q, Vaupel JW, Aviv A, McGue M (2016). DNA methylation age is associated with mortality in a longitudinal Danish twin study. Aging Cell.

[23] Perna L, Zhang Y, Mons U, Holleczek B, Saum KU, Brenner H (2016). Epigenetic age acceleration predicts cancer, cardiovascular, and all-cause mortality in a German case cohort. Clin Epigenet.

[24] Zhang Y, Saum KU, Schottker B, Holleczek B, Brenner H (2018). Methylomic survival predictors, frailty, and mortality. Aging-US.

[25] Li X, Ploner A, Wang Y, Magnusson PK, Reynolds C, Finkel D (2020). Longitudinal trajectories, correlations and mortality associations of nine biological ages across 20-years follow-up. eLife.

[26] Hillary RF, Stevenson AJ, McCartney DL, Campbell A, Walker RM, Howard DM (2020). Epigenetic measures of ageing predict the prevalence and incidence of leading causes of death and disease burden. Clin Epigenet.

[27] Dugué PA, Bassett JK, Joo JE, Baglietto L, Jung CH, Wong EM (2018). Association of DNA methylation-based biological age with health risk factors and overall and cause-specific mortality. Am J Epidemiol.

[28] Zheng C, Li L, Xu R (2019). Association of epigenetic clock with consensus molecular subtypes and overall survival of colorectal cancer. Cancer Epidemiol Biomarkers Prev.

[29] Brenner H, Kloor M, Pox CP (2014). Colorectal cancer. Lancet.

