# Supplementary material for: Whole blood DNA methylation aging markers predict colorectal cancer survival: a prospective cohort study
Source: Clin Epigenetics. 2020 Nov 30;12:184. doi: 10.1186/s13148-020-00977-4 (PMC7708179; doi:10.1186/s13148-020-00977-4)
Supplement: Supplementary file 1 — Additional file 1: Supplementary tables and figures. [file 13148_2020_977_MOESM1_ESM.pdf]

Supplementary Table 1. Levels of DNA methylation aging markers according to demographic and clinical characteristics at baseline in the DACHS study

Baseline characteristics	AgeAccelHorvath		AgeAccelHannum		DNAmMRscore		AgeAccelPheno		AgeAceelGrim	
	Median (IQR)	*P**	Median (IQR)	*P**	Median (IQR)	*P**	Median (IQR)	*P**	Median (IQR)	*P**
Sex										
Women	-1.07 (-3.56, 1.76)	<.001	-1.52 (-3.85, 1.10)	<.001	-2.03 (-2.38, -1.65)	<.001	-1.14 (-4.91, 2.53)	<.001	-3.14 (-5.31, -0.20)	<.001
Men	0.46 (-2.46, 3.19)		0.82 (-1.91, 3.35)		-1.80 (-2.15, -1.41)		0.36 (-3.48, 4.47)		1.22 (-1.45, 4.65)	
Age at diagnosis										
33 ≤ - < 55 year	-1.17 (-3.27, 2.27)	0.035	-1.00 (-3.05, 1.92)	0.064	-2.06 (-2.41, -1.68)	<.001	-0.88 (-4.51, 3.06)	0.166	-0.75 (-3.98, 3.43)	0.829
55 ≤ - < 65 year	-0.01 (-2.66, 2.79)		-0.13 (-2.64, 2.69)		-2.00 (-2.41, -1.60)		-0.22 (-3.87, 4.01)		-0.40 (-3.82, 3.54)	
65 ≤ - < 75 year	0.08 (-2.81, 2.85)		-0.03 (-2.50, 2.81)		-1.89 (-2.25, -1.50)		-0.02 (-3.96, 3.76)		-0.68 (-3.43, 3.15)	
75 ≤ - ≤ 96 year	-0.37 (-3.54, 2.72)		-0.34 (-3.45, 2.86)		-1.76 (-2.08, -1.43)		-0.57 (-4.09, 3.60)		-0.23 (-3.08, 2.35)	
TNM stage										
I	-0.16 (-2.90, 2.78)	0.229	-0.39 (-3.21, 2.13)	0.004	-2.00 (-2.34, -1.70)	<.001	-1.10 (-5.20, 2.42)	<.001	-1.17 (-4.34, 1.97)	<.001
II	-0.04 (-2.69, 2.66)		-0.03 (-2.71, 2.87)		-1.87 (-2.25, -1.47)		-0.02 (-3.65, 4.01)		-0.11 (-3.24, 3.64)	
III	-0.58 (-3.30, 2.60)		-0.71 (-3.28, 2.34)		-1.85 (-2.27, -1.48)		-0.51 (-4.29, 3.26)		-0.90 (-3.61, 2.38)	
IV	0.17 (-3.05, 2.83)		0.62 (-2.02, 3.03)		-1.80 (-2.14, -1.44)		1.12 (-2.99, 5.29)		0.46 (-2.14, 3.97)	
Charlson comorbidity index										
0 (no comorbidity)	-0.55 (-3.08, 2.48)	0.009	-0.62 (-3.19, 2.19)	<.001	-1.99 (-2.35, -1.60)	<.001	-0.97 (-4.81, 2.69)	<.001	-1.37 (-4.06, 2.22)	<.001
1 (mild comorbidity)	0.07 (-3.17, 2.98)		0.06 (-2.50, 2.95)		-1.79 (-2.16, -1.44)		0.51 (-3.15, 4.34)		0.11 (-2.53, 3.57)	
2 (moderate comorbidity)	0.20 (-2.87, 2.93)		0.47 (-2.40, 3.77)		-1.73 (-2.07, -1.39)		0.50 (-3.51, 5.24)		0.74 (-1.94, 4.18)	
3+ (severe comorbidity)	0.50 (-1.65, 3.07)		0.78 (-2.31, 4.29)		-1.66 (-2.03, -1.34)		1.64 (-2.13, 5.87)		1.39 (-1.56, 4.66)	
Tumor subsite										
Proxmal colon †	-0.45 (-3.05, 2.70)	0.436	-0.43 (-3.25, 2.44)	<.001	-1.91 (-2.30, -1.53)	<.001	-0.74 (-4.48, 3.45)	<.001	-1.12 (-3.81, 2.38)	<.001
Distal colon ‡	0.07 (-2.86, 2.82)		-0.67 (-3.25, 2.07)		-1.98 (-2.33, -1.65)		-0.95 (-4.67, 3.22)		-0.64 (-3.98, 2.54)	
Rectum	-0.31 (-3.04, 2.47)		0.59 (-2.07, 3.24)		-1.71 (-2.11, -1.35)		0.67 (-2.82, 4.46)		0.38 (-2.46, 4.19)	
BMI at diagnosis										
< 25 kg/m^2	-0.31 (-3.30, 2.59)	0.296	-0.33 (-3.01, 2.72)	0.418	-1.85 (-2.23, -1.44)	0.038	-0.41 (-4.18, 3.77)	0.750	-0.47 (-3.59, 3.64)	0.559
25 ≤ - < 30 kg/m^2	-0.31 (-2.73, 2.75)		-0.10 (-2.74, 2.81)		-1.88 (-2.26, -1.52)		-0.09 (-3.89, 3.75)		-0.46 (-3.29, 2.89)	
≥ 30 kg/m^2	-0.01 (-2.82, 2.81)		-0.44 (-2.89, 2.41)		-1.92 (-2.29, -1.56)		-0.09 (-3.93, 3.51)		-0.51 (-3.57, 2.45)	
Alcohol consumption										
Abstainer	-0.62 (-3.44, 2.22)	0.012	-0.94 (-3.32, 1.59)	<.001	-1.97 (-2.34, -1.58)	<.001	-0.69 (-4.61, 2.72)	<.001	-2.53 (-4.92, 0.63)	<.001
Female: < 20 g/day; Male: < 40 g/day	-0.23 (-2.90, 2.72)		-0.26 (-2.96, 2.70)		-1.89 (-2.26, -1.52)		-0.34 (-4.13, 3.57)		-0.42 (-3.28, 3.00)	
Female: ≥ 20 g/day; Male: ≥ 40 g/day	0.47 (-2.52, 3.56)		1.22 (-1.81, 3.72)		-1.72 (-2.09, -1.39)		1.68 (-2.52, 5.94)		2.17 (-1.27, 5.92)	
Smoking status										
Never	-0.63 (-3.15, 2.31)	0.006	-0.92 (-3.30, 2.11)	<.001	-2.03 (-2.35, -1.65)	<.001	-0.83 (-4.43, 2.75)	<.001	-2.69 (-4.87, -0.18)	<.001
Former	-0.09 (-2.83, 2.99)		0.14 (-2.61, 2.86)		-1.84 (-2.22, -1.49)		-0.20 (-3.90, 3.71)		0.58 (-2.32, 3.35)	
Current	0.25 (-2.85, 2.96)		0.01 (-2.43, 3.01)		-1.59 (-1.98, -1.21)		1.22 (-2.60, 5.77)		5.25 (1.22, 7.91)	

Abbreviation: AgeAccel, age acceleration; BMI, body mass index; DNAm, DNA methylation; TNM, tumor-node-metastasis; IQR, interquartile range; MET, metabolic equivalent of task; MRscore, mortality risk score.

**P* values are given by Kruskal-Wallis test to compare the levels of DNA methylation scores across the categorical baseline characteristics.

† The proximal colon includes the cecum, the ascending colon, and the transverse colon.

‡ The distal colon includes the descending colon (the left side of the colon) and the sigmoid colon

Supplementary Table 2. Associations between DNA methylation aging markers and all-cause mortality with comprehensive model

		Overall	Stage I & II	Stage III	Stage IV
Markers	Categories	HR (95%CI)*	HR (95%CI)*	HR (95%CI)*	HR (95%CI)*
AgeAccelHorvath	Tertile 1	1.00 (Ref.)	1.00 (Ref.)	1.00 (Ref.)	1.00 (Ref.)
	Tertile 2	0.99 (0.85, 1.15)	0.93 (0.73, 1.19)	0.94 (0.72, 1.23)	1.16 (0.83, 1.63)
	Tertile 3	1.13 (0.97, 1.32)	1.24 (0.97, 1.58)	1.13 (0.86, 1.49)	1.03 (0.75, 1.43)
	Per SD increase	1.05 (0.99, 1.12)	1.08 (0.98, 1.19)	1.10 (0.99, 1.23)	0.99 (0.86, 1.13)
AgeAccelHannum	Tertile 1	1.00 (Ref.)	1.00 (Ref.)	1.00 (Ref.)	1.00 (Ref.)
	Tertile 2	0.90 (0.76, 1.06)	0.90 (0.69, 1.17)	0.89 (0.67, 1.19)	0.79 (0.53, 1.17)
	Tertile 3	1.09 (0.92, 1.30)	1.21 (0.93, 1.56)	1.13 (0.84, 1.53)	0.93 (0.61, 1.40)
	Per SD increase	1.06 (0.98, 1.13)	1.06 (0.95, 1.19)	1.09 (0.96, 1.23)	1.03 (0.88, 1.21)
DNAmMRscore	Tertile 1	1.00 (Ref.)	1.00 (Ref.)	1.00 (Ref.)	1.00 (Ref.)
	Tertile 2	1.07 (0.88, 1.29)	1.18 (0.86, 1.61)	0.90 (0.64, 1.26)	1.11 (0.73, 1.69)
	Tertile 3	**1.61 (1.28, 2.02)**	**1.68 (1.14, 2.46)**	**1.48 (1.00, 2.18)**	**1.89 (1.16, 3.09)**
	Per SD increase	**1.36 (1.22, 1.52)**	**1.46 (1.21, 1.76)**	**1.37 (1.15, 1.65)**	**1.29 (1.02, 1.63)**
AgeAccelPheno	Tertile 1	1.00 (Ref.)	1.00 (Ref.)	1.00 (Ref.)	1.00 (Ref.)
	Tertile 2	0.98 (0.83, 1.15)	0.83 (0.64, 1.08)	0.94 (0.71, 1.25)	1.39 (0.97, 1.98)
	Tertile 3	**1.25 (1.07, 1.47)**	**1.32 (1.03, 1.70)**	1.17 (0.89, 1.55)	1.33 (0.92, 1.93)
	Per SD increase	**1.15 (1.08, 1.23)**	**1.19 (1.07, 1.31)**	**1.15 (1.02, 1.29)**	1.14 (0.97, 1.33)
AgeAccelGrim	Tertile 1	1.00 (Ref.)	1.00 (Ref.)	1.00 (Ref.)	1.00 (Ref.)
	Tertile 2	1.03 (0.92, 1.32)	1.26 (0.94, 1.70)	0.94 (0.70, 1.27)	1.03 (0.70, 1.52)
	Tertile 3	**1.69 (1.38, 2.08)**	**2.28 (1.62, 3.21)**	**1.56 (1.10, 2.22)**	1.20 (0.79, 1.81)
	Per SD increase	**1.43 (1.30, 1.56)**	**1.55 (1.35, 1.78)**	**1.19 (1.01, 1.40)**	**1.53 (1.24, 1.90)**

Abbreviation: AgeAccel, age acceleration; CI, confidence interval; DNAm, DNA methylation; HR, hazard ratio; MRscore, mortality risk score.

Numbers printed in bold: statistically significantly different from 1 ($P < 0.05$).

* Comprehensive model adjusted for age, sex, tumor stages, batch, leukocyte composition, BMI, smoking status, alcohol consumption, tumor sub-sites, and comorbidity index. Tumor stage was not included in the stage-specific analyses.

Supplementary Table 3. Associations between DNA methylation aging markers and CRC-specific mortality with comprehensive model

		Overall	Stage I & II	Stage III	Stage IV
Markers	Categories	HR (95%CI)*	HR (95%CI)*	HR (95%CI)*	HR (95%CI)*
	Tertile 1	1.00 (Ref.)	1.00 (Ref.)	1.00 (Ref.)	1.00 (ref.)
	Tertile 2	1.02 (0.83, 1.26)	0.84 (0.51, 1.39)	1.02 (0.73, 1.43)	1.15 (0.81, 1.64)
	Tertile 3	1.13 (0.92, 1.39)	1.57 (0.99, 2.51)	1.16 (0.82, 1.66)	1.01 (0.72, 1.42)
	Per SD increase	1.03 (0.95, 1.12)	1.17 (0.97, 1.41)	1.08 (0.93, 1.24)	0.96 (0.83, 1.12)
AgeAccelHannum	Tertile 1	1.00 (Ref.)	1.00 (Ref.)	1.00 (Ref.)	1.00 (ref.)
	Tertile 2	0.93 (0.74, 1.17)	1.48 (0.90, 2.45)	0.79 (0.54, 1.15)	0.82 (0.54, 1.23)
	Tertile 3	1.04 (0.82, 1.33)	1.25 (0.72, 2.16)	1.13 (0.77, 1.65)	0.92 (0.60, 1.42)
	Per SD increase	1.06 (0.96, 1.17)	1.06 (0.84, 1.32)	1.09 (0.93, 1.28)	1.05 (0.88, 1.24)
DNAmMRscore	Tertile 1	1.00 (Ref.)	1.00 (Ref.)	1.00 (Ref.)	1.00 (ref.)
	Tertile 2	0.91 (0.70, 1.19)	1.09 (0.61, 1.94)	0.85 (0.55, 1.32)	1.05 (0.68, 1.62)
	Tertile 3	**1.52 (1.12, 2.06)**	1.30 (0.64, 2.68)	**1.69 (1.02, 2.79)**	**1.73 (1.03, 2.88)**
	Per SD increase	**1.31 (1.13, 1.52)**	1.40 (0.99, 2.00)	**1.43 (1.13, 1.81)**	1.24 (0.97, 1.59)
AgeAccelPheno	Tertile 1	1.00 (Ref.)	1.00 (Ref.)	1.00 (Ref.)	1.00 (ref.)
	Tertile 2	1.22 (0.98, 1.52)	1.01 (0.62, 1.67)	1.07 (0.74, 1.54)	**1.48 (1.02, 2.13)**
	Tertile 3	**1.28 (1.02, 1.60)**	1.27 (0.76, 2.13)	1.23 (0.86, 1.77)	1.28 (0.88, 1.88)
	Per SD increase	**1.15 (1.05, 1.26)**	1.17 (0.96, 1.43)	**1.19 (1.03, 1.39)**	1.10 (0.94, 1.30)
AgeAccelGrim	Tertile 1	1.00 (Ref.)	1.00 (Ref.)	1.00 (Ref.)	1.00 (ref.)
	Tertile 2	0.95 (0.76, 1.20)	1.15 (0.67, 1.96)	0.77 (0.53, 1.13)	1.01 (0.68, 1.49)
	Tertile 3	1.25 (0.95, 1.65)	1.62 (0.83, 3.16)	1.25 (0.79, 1.97)	1.13 (0.74, 1.75)
	Per SD increase	**1.27 (1.12, 1.45)**	1.28 (0.99, 1.68)	1.10 (0.89, 1.36)	**1.48 (1.19, 1.85)**

Abbreviation: AgeAccel, age acceleration; CI, confidence interval; DNAm, DNA methylation; HR, hazard ratio; MRscore, mortality risk score.

Numbers printed in bold: statistically significantly different from 1 ($P < 0.05$).

* Comprehensive model adjusted for age, sex, tumor stages, batch, leukocyte composition, BMI, smoking status, alcohol consumption, tumor sub-sites, and comorbidity index. Tumor stage was not included in the stage-specific analyses.

Supplementary Table 4. Associations of DNA methylation aging markers with all-cause and CRC-specific mortality only among patients who did not received any chemotherapy or radiotherapy during the follow-up

		Overall survival			CRC-specific survival		
Markers	Categories	n_{death} / n_{cases}	Clinical model* HR (95%CI)	Comprehensive model[#] HR (95%CI)	n_{death} / n_{cases}	Clinical model* HR (95%CI)	Comprehensive model[#] HR (95%CI)
AgeAccelHorvath	Tertile 1	159/363	Ref.	Ref.	56/362	Ref.	Ref.
	Tertile 2	160/395	1.08 (0.85, 1.35)	1.05 (0.83, 1.34)	53/393	1.15 (0.77, 1.74)	1.16 (0.77, 1.75)
	Tertile 3	189/384	**1.30 (1.04, 1.63)**	**1.26 (1.00, 1.58)**	65/381	1.33 (0.90, 1.98)	1.37 (0.92, 2.05)
	Per 1 SD increase	508/1142	**1.11 (1.01, 1.22)**	**1.11 (1.01, 1.22)**	174/1136	1.08 (0.92, 1.26)	1.09 (0.93, 1.28)
AgeAccelHannum	Tertile 1	152/386	Ref.	Ref.	47/385	Ref.	Ref.
	Tertile 2	151/384	0.85 (0.67, 1.09)	0.84 (0.66, 1.08)	62/383	1.03 (0.68, 1.56)	0.95 (0.61, 1.45)
	Tertile 3	205/372	1.11 (0.86, 1.42)	1.16 (0.91, 1.50)	65/368	1.02 (0.65, 1.60)	1.06 (0.67, 1.69)
	Per 1 SD increase	508/1142	1.04 (0.94, 1.17)	1.07 (0.96, 1.19)	174/1136	1.05 (0.88, 1.27)	1.08 (0.89, 1.31)
DNAmMRscore	Tertile 1	110/396	Ref.	Ref.	41/395	Ref.	Ref.
	Tertile 2	176/400	**1.40 (1.06, 1.85)**	1.26 (0.94, 1.68)	57/397	1.22 (0.76, 1.97)	1.09 (0.66, 1.81)
	Tertile 3	222/346	**2.30 (1.66, 3.20)**	**1.89 (1.32, 2.69)**	76/344	**1.99 (1.14, 3.49)**	1.80 (0.98, 3.33)
	Per 1 SD increase	508/1142	**1.62 (1.40, 1.89)**	**1.50 (1.26, 1.77)**	174/1136	**1.52 (1.17, 1.97)**	**1.47 (1.10, 1.98)**
AgeAccelPheno	Tertile 1	146/402	Ref.	Ref.	46/402	Ref.	Ref.
	Tertile 2	155/386	1.02 (0.80, 1.30)	0.95 (0.74, 1.20)	68/384	1.41 (0.94, 2.12)	1.46 (0.97, 2.21)
	Tertile 3	207/354	**1.39 (1.10, 1.76)**	1.26 (0.99, 1.59)	60/350	1.17 (0.76, 1.81)	1.19 (0.77, 1.84)
	Per 1 SD increase	508/1142	**1.27 (1.16, 1.40)**	**1.24 (1.12, 1.36)**	174/1136	**1.20 (1.01, 1.43)**	**1.20 (1.00, 1.43)**
AgeAccelGrim	Tertile 1	124/414	Ref.	Ref.	52/414	Ref.	Ref.
	Tertile 2	174/386	1.28 (0.99, 1.66)	1.28 (0.97, 1.67)	56/384	1.10 (0.72, 1.69)	1.24 (0.79, 1.95)
	Tertile 3	210/342	**2.49 (1.88, 3.31)**	**2.46 (1.81, 3.36)**	66/338	**1.72 (1.07, 2.76)**	**1.99 (1.17, 3.38)**
	Per 1 SD increase	508/1142	**1.66 (1.47, 1.87)**	**1.76 (1.52, 2.03)**	174/1136	**1.43 (1.16, 1.76)**	**1.53 (1.19, 1.96)**

Abbreviation: AgeAccel, age acceleration; CI, confidence interval; HR, hazard ratio; MRscore, mortality risk score.

Numbers printed in bold: statistically significantly different from 1 ($P < 0.05$).

* Clinical model adjusted for age, sex, tumor stage, batch, and leukocyte composition.

# Comprehensive model adjusted for age, sex, tumor stage, batch, and leukocyte composition, and additionally for BMI, smoking status, alcohol consumption, tumor sub-sites, and comorbidity index.

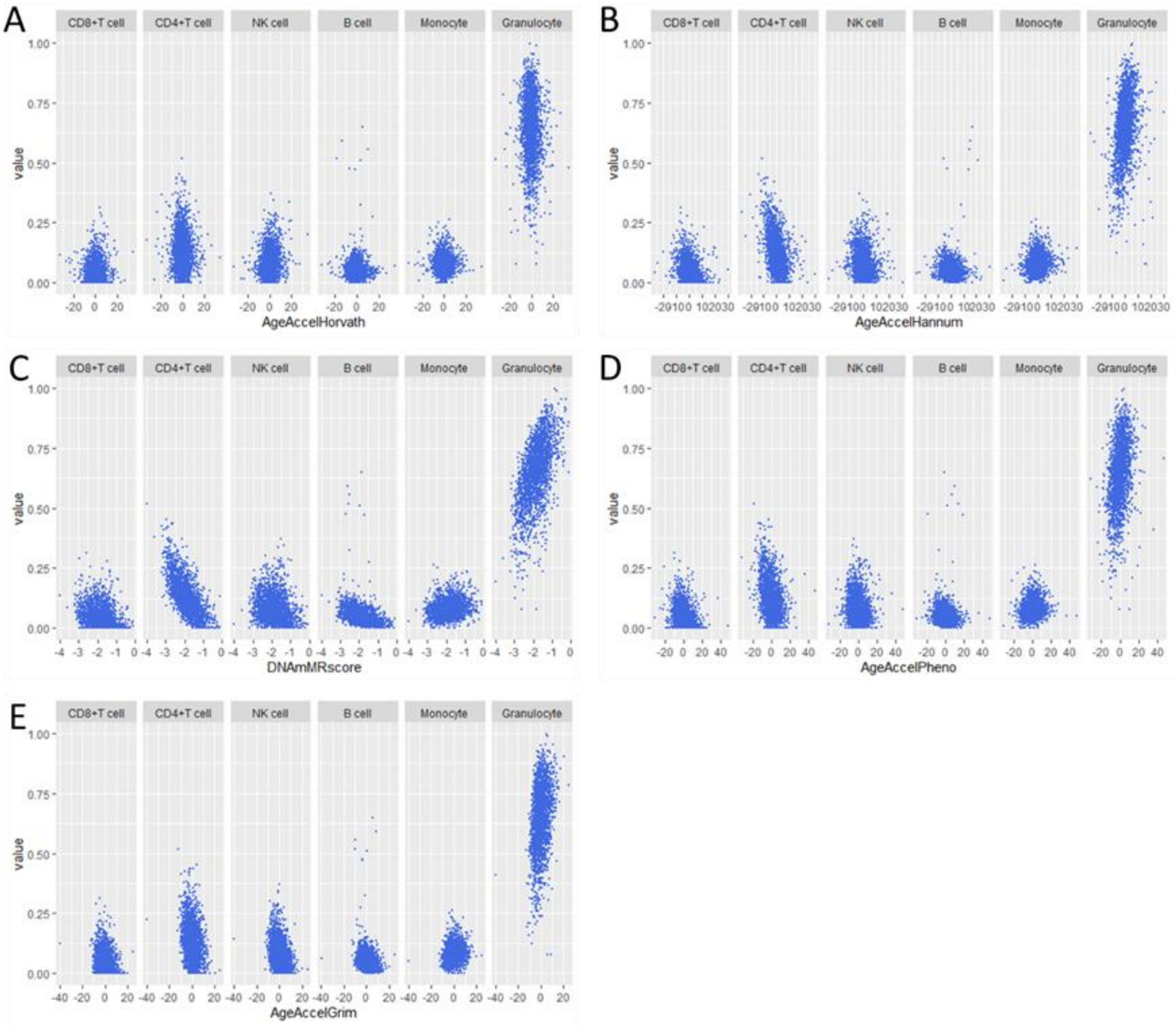

Supplementary Figure 1. Correlation of leukocyte composition with (A) AgeAccelHorvath; (B) AgeAccelHannum; (C) DNAmMRscore; (D) AgeAccelPheno; (E) AgeAccelGrim.

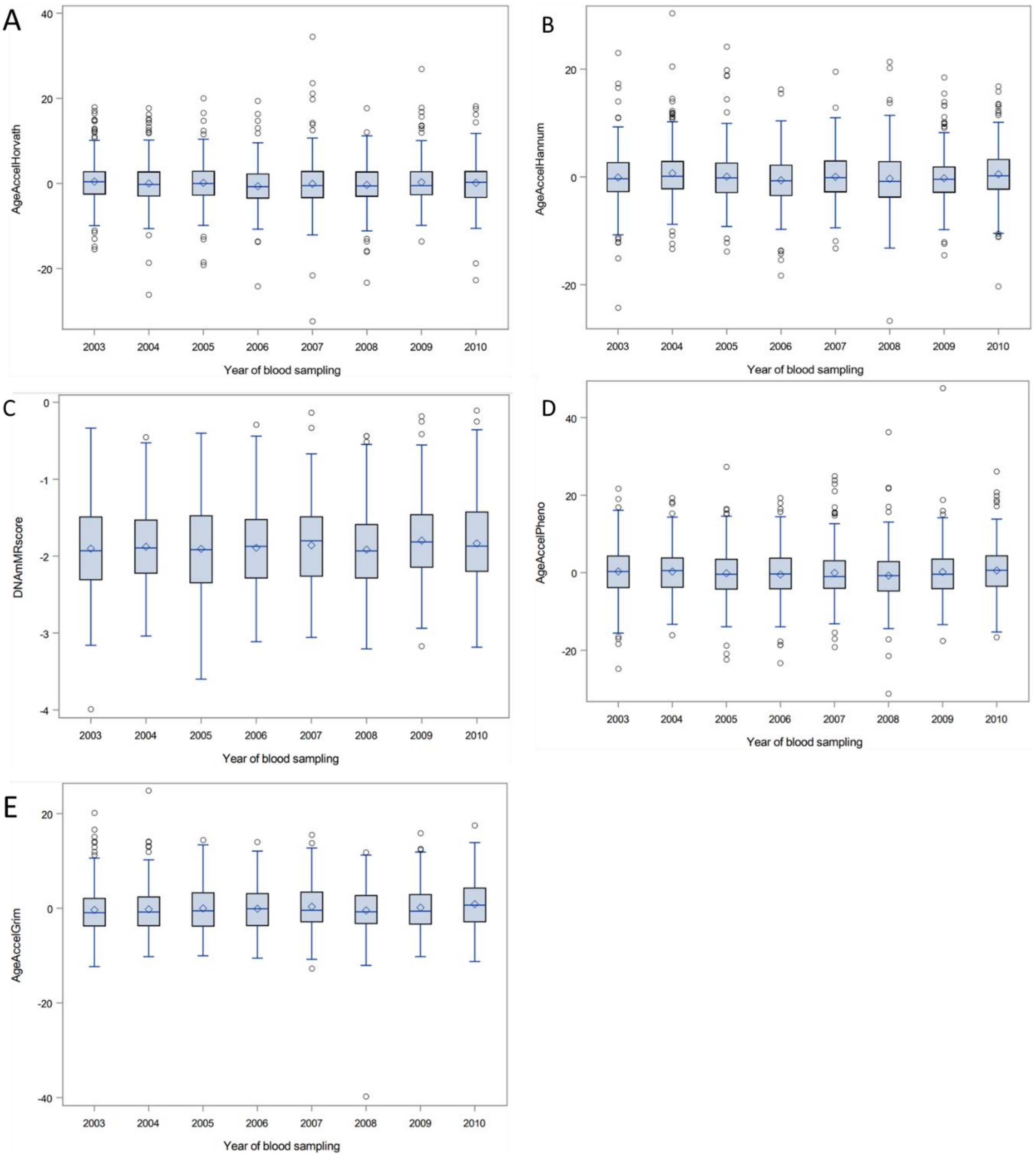

Supplementary Figure 2. Distribution of (A) AgeAccelHorvath, (B) AgeAccelHannum, (C) DNAmMRscore, (D) AgeAccelPheno and (E) AgeAccelGrim according to the year of blood sampling.

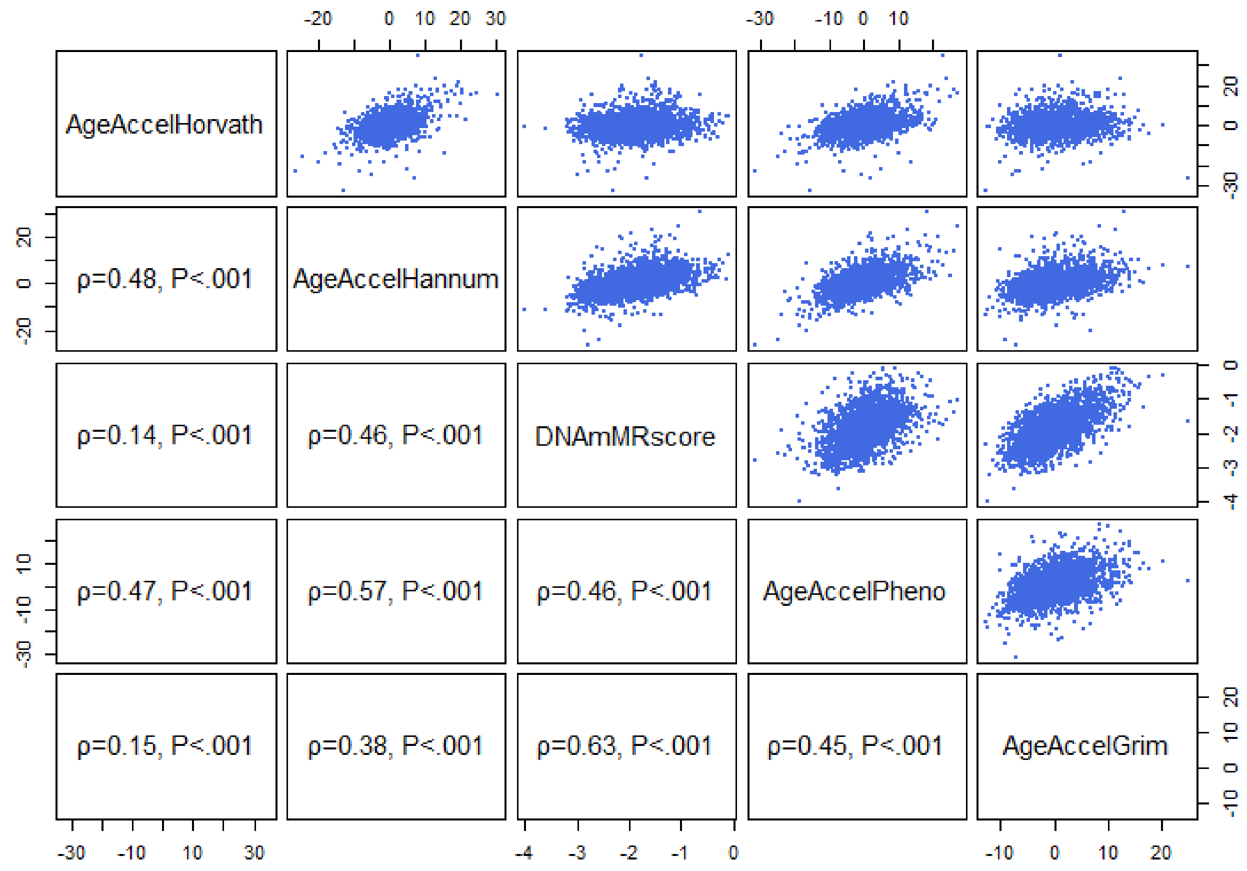

Supplementary Figure 3. Correlation matrix among the five DNA methylation aging algorithms. Numbers in the figure are Pearson correlation coefficients and *P* values. All correlation coefficients are significantly different from zero ($P < 0.001$).
